# Integrating New Prehistoric Palaeopathological Finds from Hungary

**DOI:** 10.3390/ani13121994

**Published:** 2023-06-15

**Authors:** Erika Gál, László Bartosiewicz

**Affiliations:** 1 Institute of Archaeology, Research Centre for the Humanities, Tóth Kálmán u. 4, 1097 Budapest, Hungary; 2 Osteoarchaeological Research Laboratory, Stockholm University, Lilla Frescativägen 7, 106 91 Stockholm, Sweden; laszlo.bartosiewicz@ofl.su.se

**Keywords:** dental and oral disease, arthropathy, traumatic injury, Middle Neolithic, assemblage size, classifying animal disease

## Abstract

**Simple Summary:**

The authors first present eight animal remains showing pathological changes, found in two Middle Neolithic assemblages in northern and western Hungary. Among the disorders identified in this set of animal remains, linear enamel hypoplasia was recognized for the first time in the Hungarian Neolithic. These Middle Neolithic finds were then reviewed within the context of palaeopathological data from the better-represented Early and Late Neolithic and, subsequent, (Late Copper Age and Early Bronze Age) prehistoric periods. Along with the increasing number of cases observed, patterns have begun to emerge in the taxonomic distribution of pathological lesions. The apparent great variability of the materials is, thus, discussed in terms of assemblage size, inter-observer bias and, ultimately, changes in prehistoric animal-keeping practices.

**Abstract:**

Eight newly identified pathological animal remains identified in two Middle Neolithic assemblages (ca 5330–4940 calBC) in northern and western Hungary were placed within the broader context of relevant prehistoric finds. The aim was to understand the underrepresented Middle Neolithic finds in light of the better-known cases from other prehistoric periods. The newly reported cases include arthritic and inflammatory lesions, as well as dental disorders, such as linear enamel hypoplasia, recognized for the first time in the Hungarian Neolithic. Identifications were based on bone macromorphology. When large samples are available, the frequencies of pathological bone specimens reflect the taxonomic composition. Along with the increasing number of cases, longevity related to exploitation for secondary products also became manifest. Therefore, the effects of assemblage size, disease classification and differences between authors (related to training and the time of publication) need to be considered before pathological lesions can be interpreted in terms of diachronic changes in animal husbandry.

## 1. Introduction

This paper is the first report on pathological lesions identified on Middle Neolithic animal bones in Hungary. In contrast to the much-discussed Early and Late Neolithic periods (e.g., [1,2,3]), relatively few and mostly small animal bone assemblages are known from Middle Neolithic sites. In a review of Neolithic archaeozoological assemblages from Hungary, only 7032 (a mere 7.5%) of the 93,728 identifiable bones came from this timeperiod, while the Early and Late Neolithic were represented by over 40,000 specimens each [4] (p. 52, Table 6.1). These proportions have not dramatically changed during the last two decades.

In addition to the paucity of Middle Neolithic animal bone finds from Hungary, the general rarity of pathological specimens further limits the material available to study. According to Robert W. Shufeldt, who coined the term palaeopathology, “…one may examine many hundreds of specimens before he will meet with one of their fossilized bones which shows that it was diseased at the time of the death of its owner. It is one of the very rarest of things” [5] (p. 679). It is unsurprising, therefore, that the Middle Neolithic, so far underrepresented in archaeozoological research in Hungary, is yet to be discussed in the palaeopathological literature.

Given these circumstances, aside from contributing a description of the finds to an ever-broadening base of known pathological cases, it is worth considering how they fit within the overall trends drafted on the basis of large assemblages and whether they could be interpreted between the far better-known endpoints represented by the Early and Late Neolithic and beyond.

## 2. Material and Methods

### 2.1. New Materials

**Figure 1:**
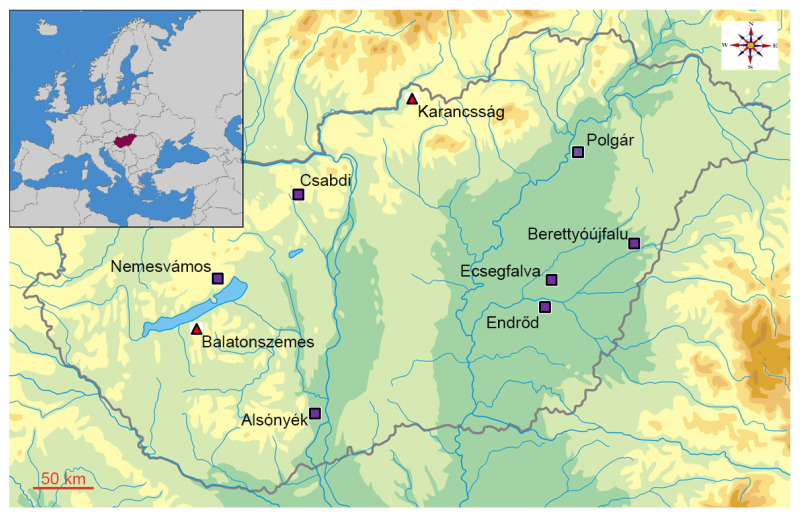
Location of the Neolithic sites mentioned in the paper. The triangles stand for the two recently studied Middle Neolithic sites, the squares indicate settlements discussed in previous studies.

The multiperiod site of Karancsság–Alsó-rétek is located in northern Hungary, near the present-day Hungarian border with Slovakia (Figure 1). Gábor Bácsmegi carried out excavations preceding the placement of drainage pipes in the area in 2002. The 1.5 m deep stratigraphy of the site revealed 9 levels of occupation and 84 archaeological features dated to various periods. Pottery remains from the Middle Neolithic features included sherds assigned to the Zseliz culture and the Music Note (*Notenkopf*) stylistic group of the Transdanubian Linear Pottery culture dated to 5330–4940 calBC. The Late Neolithic finds were assigned to the Lengyel culture and their age corresponded to 4970–4380 calBC [6] (pp. 56–61, 97). In addition to the Neolithic deposits, the remains of an early medieval (Árpád Period; 10th–11th century) settlement were found [6,7].

Among the total of 1137 animal bones found at the site, 905 could be identified at a species or (in the case of non-distinguishable elements from sheep or goats) subfamily level. The majority (67.5%) were collected from Neolithic features. The Transdanubian Linear Pottery culture (Notenkopf group) assemblage, which yielded animal bones showing pathological conditions, contained 240 identifiable specimens (NISP). The most frequently encountered species were sheep (*Ovis aries* Linnaeus, 1758) and goat (*Capra hircus* Linnaeus, 1758) at 46.7%, including non-distinguishable bones from sheep or goats (Caprinae Gray, 1821), cattle (*Bos taurus* Linnaeus, 1758) at 27.5%, and pigs (*Sus domesticus* Erxleben, 1777) at 22.1% [8] (p. 41).

The site of Balatonszemes–Bagódomb is located on the southern shore of Lake Balaton in Transdanubia, the western part of Hungary. Viktória Kiss led the preventive excavations at the site prior to the construction of the Highway M7 between 1999–2001. In total, a surface of 27,800 m^2^ was unearthed. The finds were assigned to several archaeological periods, such as the Middle Neolithic (Transdanubian Linear Pottery culture), Middle Copper Age (Balaton-Lasinja culture), the Roman Period, and the Avar Period. More than 500 features representing pits, post holes, a fragment from a log house, and inhumation graves were recovered from the central part of the Middle Neolithic settlement [9,10].

Over 7000 identifiable animal bones were found at Balatonszemes–Bagódomb, with the majority (NISP = 6748) coming from Transdanubian Linear Pottery culture features. Contrary to the coeval assemblage found at Karancsság–Alsó-rétek, at this site cattle provided most of the bones (57.6%). This species was followed in frequency by caprines (31.9%) and pigs (7.4%).

The animal bone assemblage found at Karancsság–Alsó-rétek contained only two remains displaying pathological lesions, both originating from cattle. They represented 0.8% of the identifiable Middle Neolithic bones. The larger assemblage from Balatonszemes–Bagódomb yielded six animal remains indicative of pathological conditions that made up only 0.09% of the Middle Neolithic material. Four of them originated from cattle, while two were from sheep or goats.

### 2.2. Recently Published Materials

The newly identified Middle Neolithic pathological specimens were fit within the context of the following assemblages, most of which were analyzed by ourselves in the recent past (Table 1).

Trying to limit or at least “standardize” inter-observer bias was also considered when selecting the assemblages for comparison in this paper. Inter-observer bias has always been a problem in animal palaeopathology, as it is often impossible to tell in which cases authors did not recognize or ignored pathological evidence, or whether reporting few lesions indeed reflects their rarity in the assemblage [17] (p. 357).

Four sites yielding curious individual finds are also mentioned, although these specimens could not be included in detailed analyses. They seem to have been singled out as special finds in the literature but lack the background of detailed palaeopathological analyses that would also consider other pathological finds.

### 2.3. Methods

Pathological lesions are rare, and the frequency of their reporting depends on a number of biasing factors. These include subjective elements, such as the interest and skill of the analyst, and the varying emphases placed on pathological evaluation, which make literature-based comparisons difficult [18]. Animal bones from the settlements of Balatonszemes and Karancsság have been studied by the first author. Pathological specimens were identified based on bone macromorphology, by comparison to known reference specimens. Classification also impacts on interpretation. The lesions were categorized into general groups of diseases according to Baker and Brothwell [19], and Bartosiewicz [15]. In human palaeopathology, the accuracy for specific disease recognition among several observers was 28.6%. However, correct identifications increased to 42.9% when more general categories of disease were used, making the data by different observers more comparable [20] (p. 223, Tables 1 and 2). Although similar analyses are still to be carried out in animal palaeopathology, the same trade-off between diagnostic resolution and comparability needs to be considered when relying on data published in the literature.

The size and demographic structure of prehistoric animal populations (the denominator according to which the number of pathological cases can be compared) are unknown [21] (p. 561). In want of better means of quantification, the frequency of specimens showing pathological changes can be compared to the number of identifiable specimens, a rough proxy to prevalence in archaeozoological assemblages. Estimating the minimum number of individuals (MNI), a derived variable, was not considered an adequate solution, as the remains were not found in closed features and the exact time span of deposition was not known either.

Inter-site quantitative comparisons are often hampered by extreme differences between assemblage sizes (in this case ranging between NISP = 27 to 21,692) and strong asymmetry in their distribution caused by a great number of sites with only a few diseased bones. The relationship between the two variables was, therefore, studied using their decimal logarithms to create a visually better balanced, simpler picture [22] (p. 40, Figures 2 and 3).

## 3. Results

### 3.1. Newly Identified Cases

#### 3.1.1. Dental Anomalies and Oral Pathology

**Figure 2:**
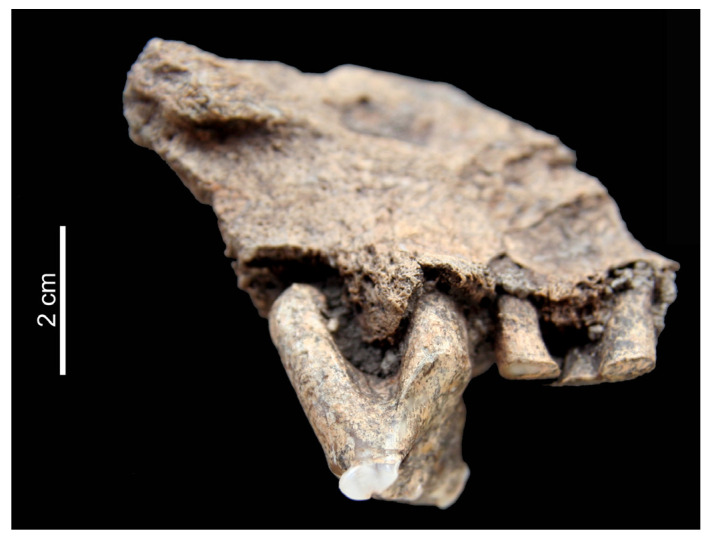
Extreme irregular tooth wear in cattle maxilla from Karancsság–Alsó-rétek (right lateral aspect).

Three of the eight pathological remains were grouped into this category. The fragment of a cattle maxilla found in Feature 37 at Karancsság–Alsó-rétek contained two teeth displaying dental deformation. The crown of the second molar is completely missing and even the root is partially worn off. The crown of the third molar seems to have been largely broken off, resulting in an extremely irregular abrasion of the remaining tooth (Figure 2). While the fragment originates from a fully grown individual, the primary cause of this deformation seems to have been trauma, as this lesion far exceeds the degree of ordinary, non-pathological tooth wear caused by age (time of exposure) and diet (abrasive agents in the food).

**Figure 3:**
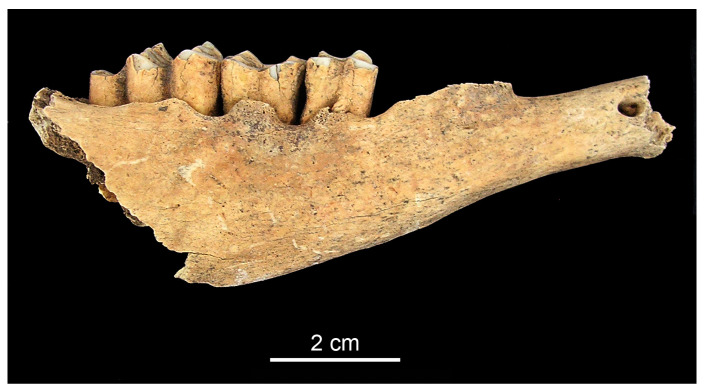
Gingivitis in a caprine mandible from Balatonszemes–Bagódomb (right lateral aspect).

Recent studies by Holmes et al. [23] have demonstrated correlations between calculus formation, alveolar recession, and new bone formation in sheep. These time (i.e., age) dependent processes lead to a chronifying stadium resulting in periodontitis. Two of these three modifications, the recession of alveoli and new bone formation could be identified on the mandible of a mature caprine found in Feature 165 at Balatonszemes–Bagódomb. The inflammation affected the osseous tissue of the mandible both between the first and second, as well as the second and third, molars (Figure 3). Similar cases were reported from various Neolithic sites [21] (p. 562, Figure 30.1).

**Figure 4:**
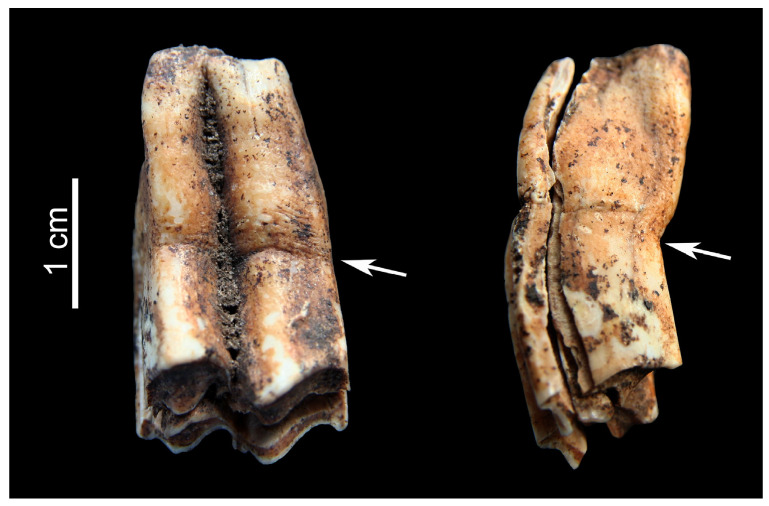
Lingual (**Left**) and aboral (**Right**) aspects of a caprine upper molar from Balatonszemes–Bagódomb showing linear enamel hypoplasia (arrows).

Linear enamel hypoplasia (LEH) is a defect in tooth formation. Since enamel does not undergo remodeling (i.e., repair), disturbances in its formation leave a permanent record in the tissue during the development of the tooth. These defects observed as lines, pits or grooves in the enamel can be used in reconstructing the individual’s life history. Enamel development and mineralization constitute a highly regulated process that can be negatively influenced by a range of systemic pathological conditions, such as fever, infection, dietary stress etc. [15] (p. 169), [24]. The intricate process of amelogenesis, governed by cells called ameloblasts, is sensitive to calcium deficiency that can cause structural deformation, mostly in the outer layer of enamel leading to hypoplasia. Enamel hypoplasia was identified in the middle crown portion of a caprine upper molar tooth recovered from Feature 11 at Balatonszemes–Bagódomb (Figure 4). A recent study regarding LEH in the molars of sheep and goats reported on its more frequent occurrence in the cervical enamel than in the middle or apical sections of the crown [24] (p. 484). Periodically arrested dental development can also be identified by extraneous cementum build-up on the root, as described in caprines from Neolithic and Chalcolithic sites in southwest Asia, tentatively linked to overgrazing and crowded keeping [25] (p. 392), [26] (p. 225).

#### 3.1.2. Arthropathies

**Figure 5:**
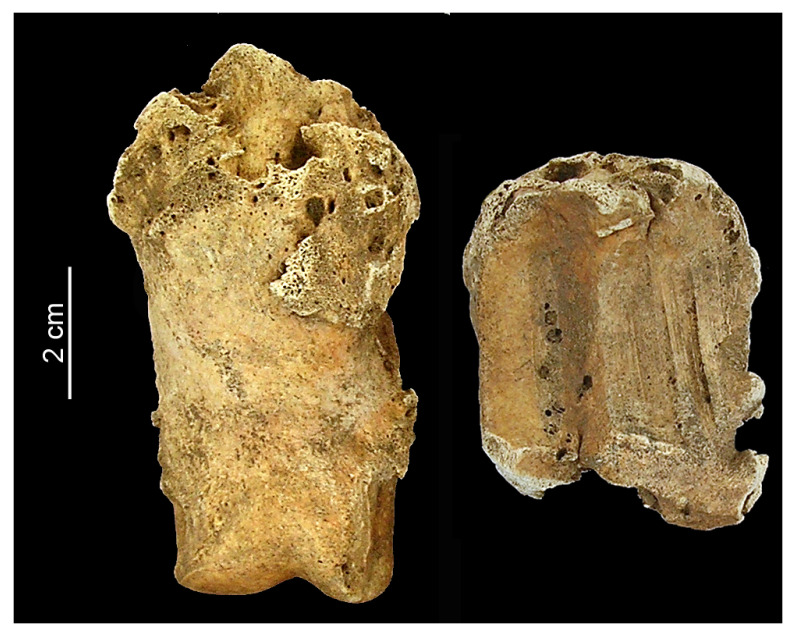
Osteophytes in the anterior aspect (**Left**), as well as eburnation and arthritic grooving on the proximal articular surface (**Right**) of the cattle proximal phalanx from Balatonszemes–Bagódomb.

The proximal phalanx originating from cattle, found in Feature 218 at Balatonszemes–Bagódomb, exhibited osteoarthritic attrition to the degree of eburnation and grooving on the proximal articular surface, and osteophyte formation around the anterior surface of the proximal end (Figure 5). These three simultaneous indicators meet most of the four criteria for osteoarthritis, as described by Baker and Brothwell [19] (p. 115). This degenerative disease primarily affects the articular cartilage in joints, but attrition can involve the bone as well. Osteoarthritis is usually attributed to overworking in cattle and horses [19] (pp. 114–117, Figure 8.9), [15] (pp. 108–109, Figures 85 and 86). Since the eburnation and grooving appeared on the proximal articular surface of the proximal phalanx from Balatonszemes–Bagódomb, the affected part was the metapodial–phalangeal joint, similar to one of the cases identified from the Roman Period settlement of Tiel-Passewaaij in the Netherlands [27] (p. 55, Figures 2 and 3). However, lesions on proximal phalanges are more likely to occur after prehistory, when the use of draught cattle became more common [28] (p. 262, Figure 7). Therefore, differential diagnoses, including secondary osteoarthritis caused by trauma, are worth considering in this case as there is no convincing evidence of cattle being used as a working animal during the Middle Neolithic.

**Figure 6:**
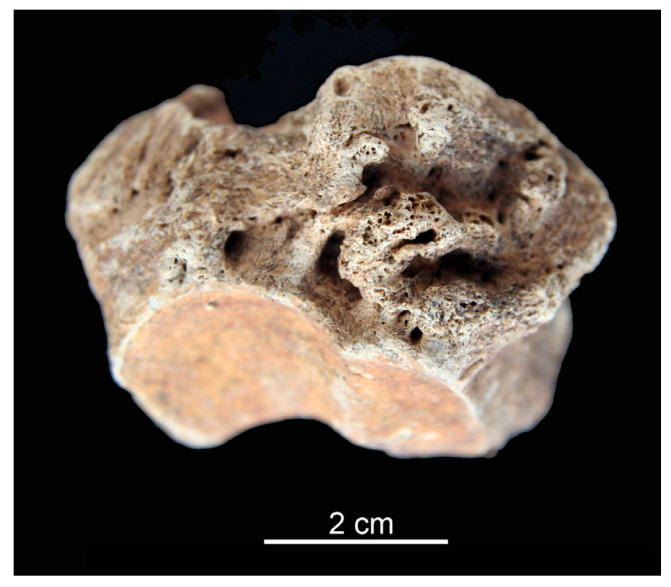
Exostosis on a bovine *os carpi radiale* from Balatonszemes–Bagódomb (medial aspect).

The medial surface of a bovine *os carpi radiale,* recovered from Feature 237 at Balatonszemes–Bagódomb, displayed a high degree of exostosis (Figure 6). The reasons for the formation of new, abnormal osseous tissue may vary, and may include the healing process for a fracture or an injury to the medial collateral ligament, inflammation due to repetitive strain (overworking), general infections, such as tertiary pulmonary diseases, metabolic diseases, and even inherited disorders [15]. Age is always worth considering behind such broad differential diagnoses, especially when chronic conditions may have been at play. Trauma, metabolic disorders, and inherited factors, however, are not directly associated with age.

#### 3.1.3. Indicators of Inflammation or Traumatic Lesion

Finally, bone remains showing light pathological lesions of unidentifiable origin were also found in both assemblages under study. A proximal fragment of cattle femur, unearthed from Feature 37 at Karancsság–Alsó-rétek, displayed signs of periostitis on the diaphysis. Two cattle rib fragments, found in Features 237 and 459 at Balatonszemes–Bagódomb, displayed uneven surfaces on the corpus. Only a rough surface to a limited extent could be observed on both pieces, a possible disturbance of the periosteum. Given the small degree of these deformations, healed fracture can be ruled out. These cases may have been caused by infectious agents or trivial injuries that did not reach the bone surface, such as those caused by smaller blows [15] (pp. 92–93). Among the differential diagnoses, malnutrition can also be considered.

### 3.2. Placing the New Finds in the Context of Published Assemblages

Pathological phenomena on animal remains from the Middle Neolithic in Hungary have not yet been described, in part due to the small number and modest size of assemblages dated to this period. On the other hand, as shown in Table 1, a number of pathological lesions were identified from three Early Neolithic (Körös culture) and two Late Neolithic (Herpály–Csőszhalom culture) settlements located in the Great Hungarian Plain, as well as an Early Neolithic (Starčevo culture) and two Late Neolithic (Lengyel and Sopot cultures, respectively) settlements in Transdanubia, western Hungary. Recently, several Late Copper Age to Middle Bronze Age bone assemblages from Transdanubia containing pathological remains have also been published [12,13,14,29]. The largest of these assemblages came from the Late Copper Age sites of Kaposújlak–Várdomb and Balatonőszöd–Temetői-dűlő.

#### 3.2.1. Early Neolithic

The assemblage unearthed at the Early Neolithic Körös culture site of Ecsegfalva 23, which contained 5494 identifiable mammalian bones, yielded seven pathological finds (0.1%). They included irregular tooth wear connected to abrasion by silica-rich grasses and soil particles ingested with the grass on a loose cattle incisor; premolar teeth lost in vivo in sheep mandible; arthritic exostosis on two sheep limb bones and a wild boar metacarpus; and deformations caused by trauma on sheep atlas and aurochs rib. While arthritic lesions on caprine bones were attributed to environmental stress endured in the marshy environment, the exostosis on the bone of wild boar, a species perfectly adapted to the humid environment, was ascribed to the old age of the individual [2] (pp. 307–308, Figures 14.13–14.15). The large assemblage (NISP = 21,692), identified from the coeval neighboring site of Endrőd 119 by Sándor Bökönyi, yielded 37 pathological specimens (0.2%). Of these, only one originated from a wild animal: a roe deer cervical vertebra that displayed a large exostosis. The other deformations covered a rather large scale of pathological conditions affecting cattle, caprines, and a dog. Most of the lesions represented healed fractures (9) and inherited disorders (7) [1] (pp. 227–233, Figures 22–26). In addition, crowded upper premolar teeth (P2–P3) in a cattle maxilla fragment were found at Endrőd 39, another Neolithic settlement near the present-day village of Gyomaendrőd [15] (p. 193, Figure 167).

**Figure 7:**
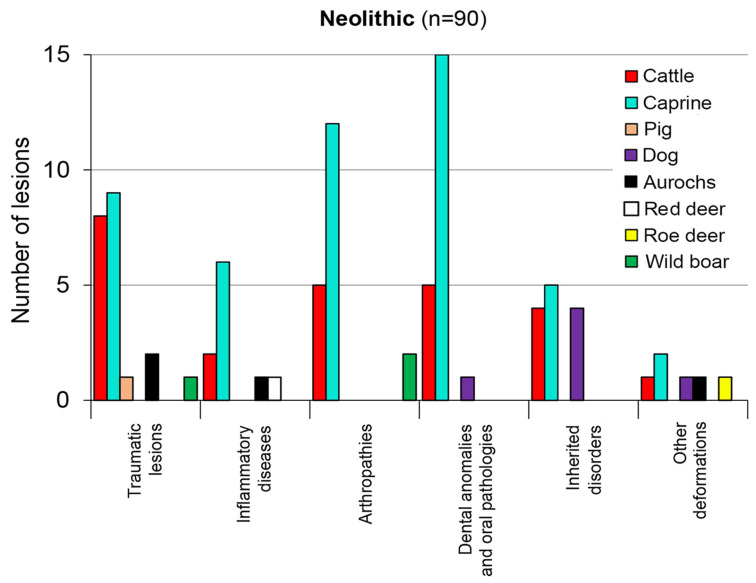
The taxonomic distribution of pathological lesions in the Neolithic assemblages.

**Figure 8:**
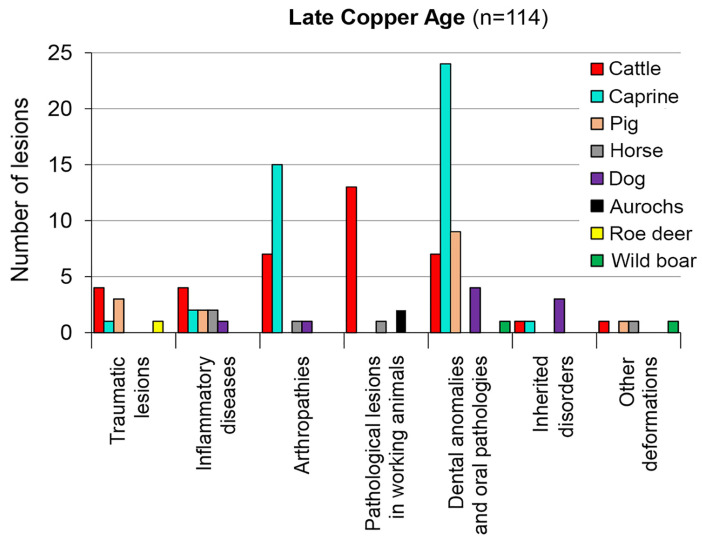
The taxonomic distribution of pathological lesions in the Late Copper Age assemblages in Transdanubia.

**Figure 9:**
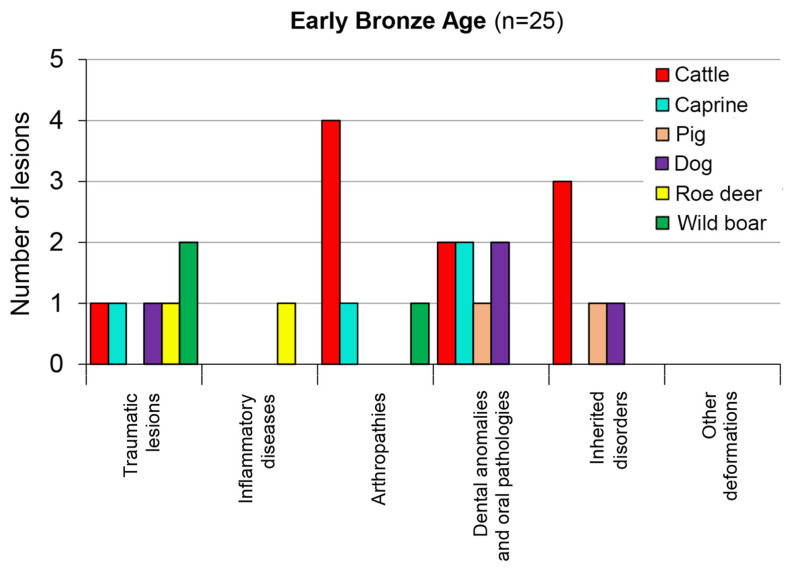
The taxonomic distribution of pathological lesions in the Early Bronze Age assemblages in Transdanubia.

Across the Danube River toward the west, the large Starčevo culture assemblage (NISP = 11,484) unearthed at Alsónyék–Bátaszék in southern Hungary yielded pathological specimens in a similar proportion (0.2%) as the aforementioned Early Neolithic materials excavated in eastern Hungary. The great majority of them (24 of 27 cases) originated from domestic animals (17 bones from caprines, 6 from cattle, and one from a dog). Wild boars were represented by two anomalous finds and a single pathological condition could be attributed to red deer. Dental diseases, such as abnormal tooth growth and irregular tooth wear (exceeding/differing from normal, age-related attrition), represented the most frequent disorders (10 cases) followed by traumatic and arthritic lesions [3] (pp. 393–397, Table 4, Figures 6–9).

#### 3.2.2. Late Neolithic

Pathological lesions found in Late Neolithic assemblages are sometimes represented by individual finds singled out for publication, without additional pathological information on the assemblage. They include a flint blade wedged into the caudal articular surface of the atlas from aurochs (Polgár–Csőszhalom) [16] (p. 104, Figure 4); a tuberculous cavern on the diaphysis of a cattle metatarsus (Berettyóújfalu–Herpály) [15] (p. 101, Figure 81); a deformed elbow joint due to a healed compound fracture with massive callus in cattle (Csabdi–Télizöldes) [15] p. 210, Figure 181); and crowded teeth in a cattle maxilla (Endrőd 39) [15] (p. 193, Figure 167).

In addition, a total of six pathological lesions were identified from a number of Late Neolithic Lengyel culture assemblages unearthed at Alsónyék–Bátaszék, including two large pits with household refuse [30]. A cattle mandible developed uneven tooth wear on the P2–P3 and a cattle proximal phalanx with exostosis was also found. The metacarpus of an aurochs displayed intense exostosis on its medio-distal part, most likely due to mechanical trauma. The other pathology from this species appeared in the form of bone growth on the diaphysis of a humerus. Alsónyék–Bátaszék also yielded pits with dog skulls, and dog burials [31] (p. 68). The dog skeleton in Feature 723 displayed oligodonty missing the first premolar (P1) in the left maxilla. Finally, the dog cranium No 3 in Feature 69 seems to have contained a rostrally deviated upper canine tooth [31] (p. 60, Figure 33). This condition is caused by the retention of the deciduous tooth, which tilts the erupting permanent canine tooth into an abnormal position. As a result, the opposing canine may not have room to occlude properly. The misdirected canine syndrome may cause abnormal tooth wear, periodontal disease, or even early tooth loss [32].

In contrast with the assemblages unearthed at Karancsság–Alsó-rétek and Balatonszemes–Bagódomb, respectively, that yielded more cattle than caprine remains displaying pathology, the summary of results from the discussed Neolithic sites indicated a higher frequency of sheep and goats for all categories of lesions (Figure 7). This result is unsurprising in the case of the well-represented Early Neolithic assemblages from Endrőd 119 and Ecsegfalva 23, in which caprines dominated by 70.8% and 68.9%, respectively. Sheep and goat keeping seems to have been a culturally determined, strong tradition among the Early Neolithic pastoral communities, presumably of Balkan origins, who established Körös culture settlements in Hungary. However, caprines of Asian origin were adapted to more arid grassland environments. They were, thus, probably exposed to environmental stress in the marshy habitat of the Great Hungarian Plain [2] (pp. 310–311). On the other hand, the striking frequency of tooth malformations observed in sheep and goat finds at the coeval Starčevo culture site of Alsónyék-Bátaszék in southern Hungary, where caprines and cattle seem to have been equally represented (4688 and 4633 remains, respectively) [3] (p. 390, Table 1), may point to poor quality forage.

The pooling of results for the Neolithic period in Figure 7, also adds emphasis to the presence of arthropathies, making this group of deformations the second most frequent pathology after the traumatic lesions, as well as dental anomalies and oral pathologies (each represented by 21 affected specimens), among the total of 90 cases.

#### 3.2.3. Late Copper Age and Early Bronze Age

Recently, several Late Copper Age to Middle Bronze Age bone assemblages have been published from northern and southwestern Transdanubia that also contained a number of pathological remains [12,13,14,29].

The Late Copper Age settlements yielded 114 pathological lesions. Caprines provided the greatest number of remains (43) during this time, with the majority representing joint diseases (15), as well as dental and oral pathologies (24). Cattle furnished 37 pathological finds, becoming the leading species concerning pathological phenomena commonly associated with working animals in the literature that may be linked with the use of draught cattle (Figure 8). The increased likelihood of Copper Age draught exploitation is shown by the metapodial asymmetry in cattle at the site of Balatonőszöd. This deformation develops in response to excess loading [33] (p. 43). Therefore, it may also rarely occur in large aurochs bulls as in the case of two specimens from the same site [12] (p. 315, Figure 232.2). The few dog remains mostly displayed dental and oral pathologies, including congenital tooth disorders.

The majority of the Late Copper Age remains were identified from the sites of Kaposújlak–Várdomb and Balatonőszöd–Temetői-dűlő, respectively, among which dental anomalies and oral pathologies (uneven tooth wear and abnormal tooth roots linked with chronic infection) [19] (pp. 150–151, Figure 9.a), as well as arthropathies, were the most common (24 and 45 cases, respectively). Although the remains of caprines dominated over those of cattle at both sites, cattle bones showed more pathological lesions than the remains of sheep and goats at Balatonőszöd (Table 2), [12] (pp. 312–316, Figures 229–233), [13] (pp. 19–20, Figure 6).

In spite of the greater number of assemblages identified from the Early Bronze Age in Transdanubia [13] (p. 63, Figure 27), only the two largest yielded remains with pathological cases (a total of 25 from Kaposújlak–Várdomb and Paks–Gyapa, see Table 1). Cattle were the most common species in this period, often kept until old age. As a result, this species yielded the majority of pathological finds. In addition to its top position among arthritic lesions, cattle are the leading species in the group of inherited disorders as well. While the Early Bronze Age assemblage is of modest size in comparison with the previously discussed two prehistoric periods, it is notable that sheep and goats continuously seem to have been prone to dental damage and oral diseases (Figure 9). This trend in Europe holds true, even in comparison with caprine morbidity data from prehistoric sites in southwest Asia, where arthropathies of caprines seem to be more common than dental disorders [34].

## 4. Discussion

Although one may assume that large assemblages yield more pathological cases in proportion with size, the percentages of lesions listed in Table 1 strongly vary among the studied sites. In her review of 18 British sites, Jane Siegel [17] (p. 358, Table 2) reported 0.24% pathological specimens on the basis of 47,300 bones from 18 sites in Britain. In the meantime, a review of 260,475 specimens from 18 sites [35] (p. 257) yielded only a 0.029% ratio of bones with pathological lesions. A recent review of 128 assemblages representing 3,253,632 specimens contained 0.406% pathological cases [15] (p. 35). The reasons behind this variability are complex. The only cemetery material from Pilismarót is an understandable outlier with 7.14%, as its taphonomy is completely different from those of settlement assemblages: it reflects a concentration of pathological information within a very small assemblage of potentially articulated bones, i.e., individual skeletons. The rest of the values rarely reach 0.5%, falling largely close to the aforementioned values established in the three major surveys.

**Figure 10:**
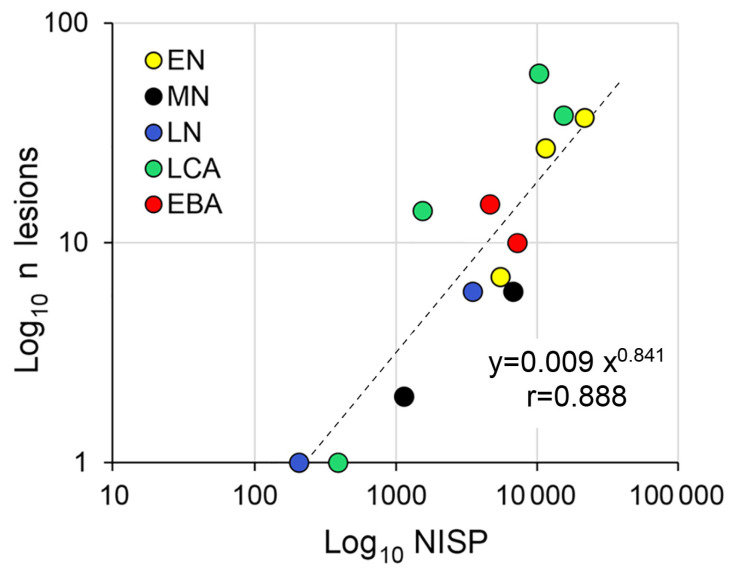
The relationship between assemblage size (NISP) and the number of pathological lesions in the material available for study. Legend: EN = Early Neolithic; MN = Middle Neolithic; LN = Late Neolithic; LCA = Late Copper Age; EBA = Early Bronze Age.

However, being ratio values, percentages disregard assemblage size and are notoriously inaccurate in characterizing phenomena whose distribution is other than Gaussian [36]. In fact, 28 of the aforementioned 128 assemblages contained only a single pathological bone, and only low percentages of pathological cases were associated with found materials exceeding 300 identifiable specimens [15] (p. 34, Table 2). This shows that NISP always needs to be taken into consideration in the evaluation of pathological cases. In Figure 10, the number of pathological specimens was plotted against NISP in the settlement materials under discussion here. Singular Neolithic finds listed at the bottom of Table 1 and the Pilismarót funerary context were not included in this comparison. Transformation to decimal logarithms was used to produce a linear presentation, for easier visual appraisal.

As shown by the high, positive correlation in Figure 10, the number of pathological finds understandably increases along with assemblage size. However, the relationship itself is not completely linear, looking slightly degressive (the exponent of x is only 0.841, rather than 1 in the figure). This means that, in the studied data set, increasing assemblage size does not necessarily result in a proportionally higher number of pathological finds.

The relation of data points in this figure shows that larger (Early Neolithic) and more recently studied (Late Copper Age) assemblages tend to have relatively more pathological cases, as they fall above the trend line. Similarly, high proportions of pathological bone in large assemblages tend to occur in recent publications, usually prepared by more experienced authors (e.g., [37,38]), reflecting an increased awareness of palaeopathology. In more recent analyses, increasing attention has been paid to pathological lesions.

Beyond these general trends, however, individual animal species need to be understood separately in each period, as shown in Figure 7, Figure 8 and Figure 9. In spite of random sampling bias and inter-observer differences, patterns in animal morbidity also need to be identified. Species composition, through the mediation of different forms of animal exploitation expressed in different age profiles, has a direct influence on the manifestation of animal disease in archaeological assemblages.

During the Early Neolithic and the Late Copper Age, caprines played an important role, thus their pathological finds also tend to dominate. Pathological changes are rarely manifest in the remains of wild animals. While natural selection understandably acted against diseased individuals in the absence of human care, pathological lesions (especially trauma [39]) tend to become more visible in game animals at sites where their remains are represented in higher numbers in the food refuse (e.g., Late Neolithic and Late Copper Age settlements).

Older animals have more time and, thus, a greater statistical probability of developing one or more lesions on their bones than young individuals. Therefore, if domestic animals were preferentially killed at an early age, the ‘herd’ may look healthy [17]. Pigs, usually slaughtered for their meat when young, tend to improve the overall health statistics. On the other hand, the emergence of secondary (that is, renewable) products, such as milk, wool or draught power, increases the longevity of animals repeatedly exploited in these ways. In addition to pathological conditions potentially caused by animal use itself, the larger body mass and older age of such animals increases morbidity in later prehistoric periods (Figure 8 and Figure 9).

## 5. Conclusions

Pathological phenomena are manifested in archaeozoological assemblages through the filters of prehistoric animal exploitation and meat consumption, specifically. The eight Middle Neolithic bones showing pathological lesions first published in this paper fit within the three most commonly observed groups of osteologically manifested diseases in the Neolithic, such as traumatic lesions, arthropathies, and dental/oral pathologies. This is a clear reflection of the law of large numbers, as the most frequently occurring pathological conditions are most likely to be manifested even in very small samples.

In the absence of other sets of Middle Neolithic pathological lesions in the Hungarian archaeozoological literature, the new finds were integrated within the framework of a selected set of prehistoric assemblages in order to facilitate the drawing of broader diachronic conclusions. Traumatic lesions in livestock may have been caused primarily by humans or sometimes by abiotic environmental accidents. Among the dental anomalies, linear enamel hypoplasia, a defect linked to dietary or other stress factors, was first identified in a Neolithic animal in Hungary.

In the two recently studied Middle Neolithic assemblages, six of the eight animal bones displaying traumatic lesions belonged to cattle. In contrast with the Early Neolithic and Late Copper Age when sheep dominated, cattle seem to have yielded a larger number of pathological lesions in the Late Neolithic materials, too. By the Late Copper Age and the beginning of the Early Bronze Age, when cattle longevity increased likely due to draught use in transport and tillage, the contribution of cattle bones to the set of pathologically modified remains becomes even more apparent.

## Figures and Tables

**Table 1:** Prehistoric assemblages used in this study. Abbreviations: EN = Early Neolithic; MN = Middle Neolithic; LN = Late Neolithic; LCA = Late Copper Age; EBA = Early Bronze Age.

Age	Site	NISP Total	N Pathology	Pathological %	Reference
EN	Alsónyék	11,484	27	0.24	[3]
EN	Ecsegfalva 23	5494	7	0.13	[2]
EN	Endrőd 119	21,692	37	0.17	[1]
MN	Balatonszemes	6748	6	0.09	This paper
MN	Karancsság	1137	2	0.18	This paper
LN	Alsónyék	3490	6	0.17	This paper
LN	Nemesvámos	205	1	0.49	[11]
LCA	Balatonőszöd	15,400	38	0.25	[12]
LCA	Kaposújlak	10,285	59	0.57	[13]
LCA	Paks	387	1	0.26	[13]
LCA	Pilismarót	28	2	7.14	[14]
LCA	Szűr	1537	14	0.91	[13]
EBA	Kaposújlak	4625	15	0.32	[13]
EBA	Paks	7238	10	0.14	[13]
Pathological specimens singled out for publication					
EN	Endrőd 39	-	1	-	[15]
LN	Berettyóújfalu	392	1	-	[15]
LN	Csabdi	852	1	-	[15]
LN	Polgár	5823	1	-	[16]

**Table 2:** The proportion of pathological cases/NISP in two major Late Copper Age assemblages.

Site	Culture	Cattle	Caprine
Balatonőszöd	Boleráz and Baden	17/5879	8/6066
Kaposújlak	Baden	15/1765	31/6683

## Data Availability

All data used in the current study are available from the corresponding author upon reasonable request.
